# Antimicrobial Potential of Pomegranate and Lemon Extracts Alone or in Combination with Antibiotics against Pathogens

**DOI:** 10.3390/ijms25136943

**Published:** 2024-06-25

**Authors:** Grace Farhat, Lewis Cheng, Emad A. S. Al-Dujaili, Mikhajlo Zubko

**Affiliations:** 1 Faculty of Health and Education, Manchester Metropolitan University, Manchester M15 6BG, UK; 2 Centre for Cardiovascular Science, Faculty of Medicine and Veterinary Medicine, Queen’s Medical Research Institute, University of Edinburgh, Edinburgh EH16 4TJ, UK; 3 Faculty of Science and Engineering, Manchester Metropolitan University, Manchester M15 6BH, UK

**Keywords:** pomegranate extract, lemon extract, antibiotic resistance, antioxidants, anti-microbial activity

## Abstract

Amidst the growing concern of antimicrobial resistance as a significant health challenge, research has emerged, focusing on elucidating the antimicrobial potential of polyphenol-rich extracts to reduce reliance on antibiotics. Previous studies explored the antifungal effects of extracts as potential alternatives to conventional therapeutic strategies. We aimed to assess the antibacterial and antifungal effects of standardised pomegranate extract (PE) and lemon extract (LE) using a range of Gram-negative and Gram-positive bacteria and two yeast species. Additionally, we assessed the antimicrobial activities of common antibiotics (*Ciprofloxacin*, *Imipenem*, *Gentamicin*, and *Ceftazidime*), either alone or in combination with extracts, against *Staphylococcus aureus* and *Escherichia coli*. PE displayed substantial antibacterial (primarily bactericidal) and antifungal effects against most pathogens, while LE exhibited antibacterial (mostly bacteriostatic) and antifungal properties to a lesser extent. When compared with antibiotics, PE showed a greater zone of inhibition (ZOI) than *Ciprofloxacin* and *Ceftazidime* (*p* < 0.01) and comparable ZOI to *Gentamicin* (*p* = 0.4) against *Staphylococcus aureus*. However, combinations of either PE or LE with antibiotics exhibited either neutral or antagonistic effects on antibiotic activity against *Staphylococcus aureus* and *Escherichia coli*. These findings contribute to the existing evidence regarding the antimicrobial effects of PE and LE. They add to the body of research suggesting that polyphenols exert both antagonistic and synergistic effects in antimicrobial activity. This highlights the importance of identifying optimal polyphenol concentrations that can enhance antibiotic activity and reduce antibiotic resistance. Further in vivo studies, starting with animal trials and progressing to human trials, may potentially lead to recommendation of these extracts for therapeutic use.

## 1. Introduction

Antimicrobial resistance remains a significant public health challenge, compromising the efficacy of treatments and contributing to prolonged hospitalisations and escalating healthcare expenditures [1]. Approximately USD 20 billion is added to the annual healthcare expenditure in the United States due to antimicrobial resistance [2]. With antibiotic resistance being primarily caused by unnecessary antibiotic use, there is an urgent need to reduce consumption and reduce mortality and delay in recovery [3]. The “threat list” of pathogens includes common Gram-negative bacteria (*Pseudomonas aeruginosa* (*P. aeruginosa*), *Klebsiella pneumoniae* (*K. pneumoniae*), *Escherichia coli* (*E. coli*)), and Gram-positive bacteria (*Staphylococcus aureus* (*S. aureus*) and MRSA (methicillin-resistant *Staphylococcus aureus*)) [4]. These strains can become resistant to antibiotics through multiple mechanisms [5]. Infections caused by *Klebsiella oxytoca* (*K. oxytoca*), *Bacillus cereus* (*B. cereus*), and *Staphylococcus epidermidis* (*S. epidermidis*) are less common; nonetheless, they have the potential to cause serious infections and antibiotic resistance [6,7,8]. Additionally, fungal strains can be detrimental to human health [9], with *Candida albicans* (*C. albicans*) and *Candida glabrata* (*C. glabrata*) being considered the most pathogenic yeasts in humans [10]. 

To effectively address the challenges of antibiotic resistance, it becomes imperative to seek alternatives to current antibiotics. Over the past two decades, numerous plant-derived compounds have been studied for their antioxidant and antimicrobial potential. These properties have often been attributed to their polyphenol content [11]. Notably, cranberry juice, rich in flavonoids and phenolic acids, has been proposed as a potential total or partial therapeutic alternative to antibiotics in the treatment of urinary tract infections [12]. Other polyphenol-rich foods, like curcumin, garlic, cumin, turmeric, and ginger, exhibited antibacterial activity against common microbial strains, and have also shown antifungal effects [11].

Pomegranate (*Punica granatum*) and lemon (Citrus lemon) are two environmentally sustainable plants with high levels of flavonoids and phenolic acids [13,14]. Pomegranate is rich in ellagitannins (a subtype of phenolic acid), particularly punicalagins, as well as anthocyanins [15], while lemon is a source of eriocitrin (a subtype of flavanone) [16]. Pomegranate in fruits, juices, peels, and/or extracts has shown significant potential in the inhibition of several bacterial species (notably *E. coli*, *P. aeruginosa*, *S. aureus*, and MRSA) and fungal species (*C. albicans*, *C. glabrata*) [17,18]. Similarly, fresh lemon, lemon juice, lemon peel, and lemon extract have been reported to have antibacterial and antifungal effects [19,20]. Nevertheless, the use of diverse non-standardised extracts rendered it more complicated to compare results across studies. 

Numerous studies looked at whether polyphenols can enhance the therapeutic effects of antibiotics. They mostly reported synergistic effects when combining extracts and antibiotics, suggesting their potential in either partial or complete replacement of antibiotics [20,21,22]. Validating such effects, while expanding the testing of different antibiotics and a range of extracts with known concentration of polyphenols, will contribute to considering their use in combined therapy in humans. Our study, therefore, aimed to achieve the following: (a) assess the antibacterial and antifungal effects of standardised lemon extract (LE) and pomegranate extract (PE) against prevalent Gram-positive bacteria (*B. cereus*, MRSA, and *S. epidermidis*), Gram-negative bacteria (*E. coli, K. oxytoca, K. pneumoniae*, and *P. aeruginosa*), and yeasts (*C. albicans* and *C. glabrata*), as well as their mode of inhibition; (b) evaluate the antimicrobial activity of common antibiotics (*Ciprofloxacin*, *Imipenem*, *Gentamicin*, and *Ceftazidime*) and the extracts, either individually or combined, against two of the most common bacterial pathogens (*S. aureus* and *E. coli*). The findings may serve as a basis for further in vivo research exploring the effectiveness of these natural compounds in lowering antibiotic resistance and combating infections.

## 2. Results

### 2.1. Antimicrobial Effects of Lemon and Pomegranate Extracts

#### Sensitivity Testing and Modes of Inhibition

ZOI results are presented in Supplementary Materials S1. PE inhibited the growth of *B. cereus* (ZOI: 16.67 ± 1.15 mm), MRSA (ZOI: 26.67 ± 1.15 mm), *E. coli* (ZOI: 13.33 ± 1.15 mm), *P. aeruginosa* (ZOI: 18.67 ± 1.15 mm) and *C. albicans* (ZOI: 13.33 ± 1.15 mm), *K. oxytoca* (ZOI: 12 ± 0 mm), *S. epidermidis* (ZOI: 12 ± 0 mm), and *C. glabrata* (16 ± 0 mm). No zones of inhibition were observed for *K. pneumoniae*. 

**Figure 1:**
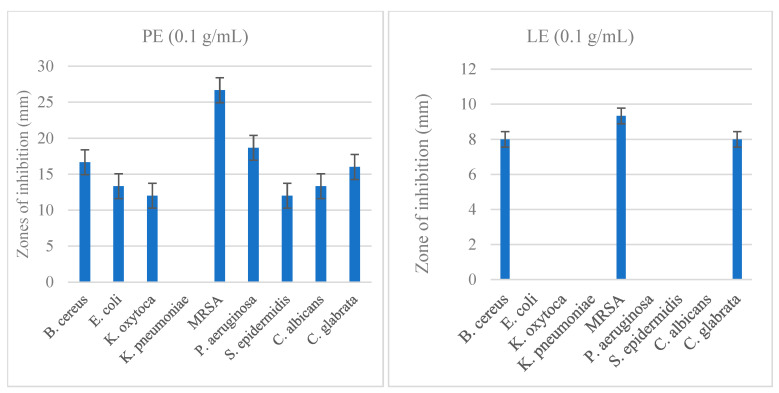
Antibacterial and antifungal activities of pomegranate and lemon extracts in disc diffusion assays. Values are expressed as mean ZOI (SEM); MRSA: methicillin-resistant *Staphylococcus aureus.*

LE was effective in inhibiting *B. cereus* (ZOI: 8 ± 0 mm) and MRSA (ZOI: 9.33 ± 1.15 mm), but to a lesser extent than PE. ZOI for *C. glabrata* was reduced with LE (ZOI: 8 ± 0 mm) (Figure 1).

With regards to modes of inhibition, bactericidal effects were revealed for PE against *B. cereus*, *K. oxytoca,* MRSA, and *P. aeruginosa*, as well as for LE against *B. cereus*. Bacteriostatic effects were observed for PE against *E. coli* and *S. epidermidis*, and for LE against MRSA. Additionally, PE exhibited fungistatic effects against *C. albicans* and *C. glabrata*, while LE showed similar effects against *C. glabrata*. No fungicidal effects were observed (Supplementary Materials S1).

### 2.2. Anti-Microbial Activities of Extracts and Antibiotics Used Separately and in Combination

#### 2.2.1. Impact of Antibiotics, PE, and LE Extracts on the Growth of *S. aureus*

All images of plates are presented in Supplementary Materials S2. A 20 µL solution of PE (0.1 g/mL stock) resulted in greater ZOI for *S. aureus* (22.58 ± 0.8 mm) than 20 µL solutions of *Ciprofloxacin* (ZOI: 17 ± 3.54 mm, *p* = 0.002) and *Ceftazidime* (ZOI: 9 ± 0.25 mm, *p* < 0.001). No significant differences in ZOI were noted for solutions of 20 µL of *Gentamicin* (ZOI: 20.5 ± 2.17 mm) and either of the two solutions of PE (*p* = 0.4). However, PE exerted greater zones of inhibition compared to a lower concentration of *Gentamicin* (10 µL solution) (*p* = 0.006). *Imipenem* strongly inhibited the growth of *S. aureus* after application of both volumes 10 µL (ZOI: 34.5 ± 1.25 mm) and 20 µL (ZOI: 37 ± 5.68 mm), both zones of inhibition being significantly larger than for those for 10 and 20 µL PE solutions (*p* < 0.001). 

**Figure 2:**
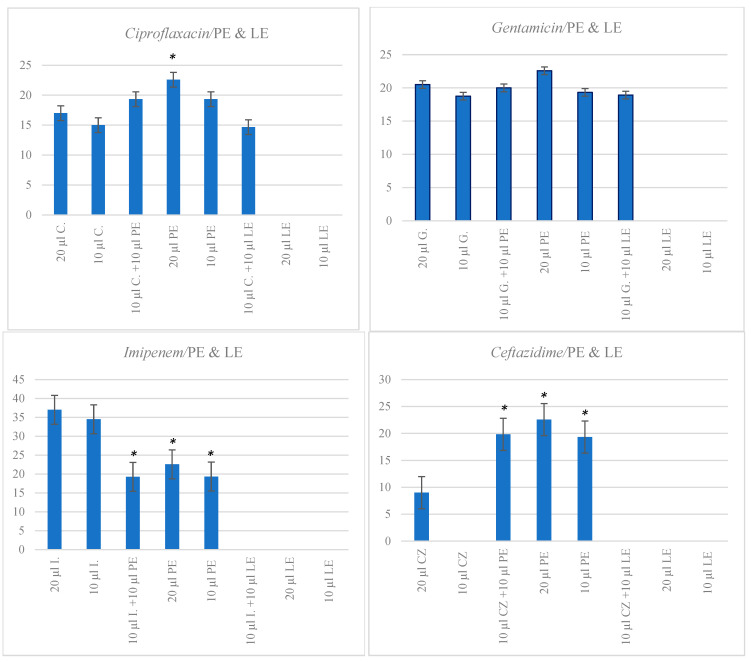
Zones of inhibition of *S. aureus* growth by antibiotics and extracts at various concentrations. Values are expressed as mean ZOI (SEM). *y*-axis represents zones of inhibition (ZOI); C. Ciprofloxacin; G: Gentamicin; I: Imipenem; CZ: Ceftazidime. * *p* < 0.001. Significance is compared to that in assays involving 20 µL solution of antibiotics.

As for LE (10 µL and 20 µL solutions at 0.1 g/mL), it did not inhibit bacterial growth and led to negligible ZOI of *S. aureus* (Figure 2).

#### Combinations of Extracts and Antibiotics

When 10 µL of PE solution was added to 10 µL of *Ciprofloxacin*, it resulted in a small but significant increase in ZOI size for *S. aureus* (15 ± 1.56 mm) when compared to the antibiotic alone (ZOI: 19.33 ± 5.2 mm, *p* = 0.03). No significant alterations in ZOI were observed for *Ceftazidime* (10 µL) or *Gentamicin* when PE (10 µL) was added to both solutions (*p* > 0.05). Nevertheless, in contrast to a 10 µL *Imipenem* solution alone, the combination of 10 µL of PE and 10 µL *Imipenem* solution led to a substantial decrease in the ZOI of *S. aureus* (*p* < 0.001). 

When LE was combined with *Ciprofloxacin*, *Gentamicin*, or *Ceftazidime*, there were no significant differences in ZOI size compared to the respective antibiotics alone (*p* > 0.05). However, the combination of 10 µL of *Imipenem* with 10 µL of LE resulted in a significant reduction in ZOI size compared to *Imipenem* alone (*p* < 0.002) (Figure 2).

#### 2.2.2. Impact of Antibiotics and Extracts on the Growth of *E. coli*

**Figure 3:**
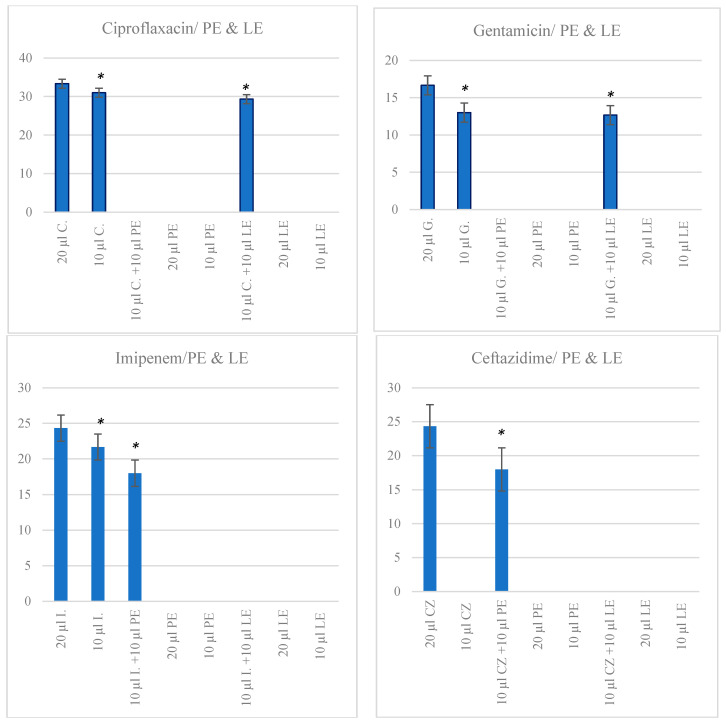
Zones of inhibition of *E. coli* growth by antibiotics and extracts at various concentrations. Values are expressed as mean ZOI (SEM). *y*-axis represents zones of inhibition (ZOI); C: Ciprofloxacin; G: Gentamicin; I: Imipenem; CZ: Ceftazidime. * *p* < 0.001. Significance is compared to that in assays involving 20 µL solution of antibiotics.

Images from plates are presented in Supplementary Materials S3. PE and LE exerted marginal or no zones of inhibition on *E. coli* and had no impact on the growth of this microorganism (Figure 3).

#### Combinations of Extracts and Antibiotics

The addition of 10 µL of LE solution to either *Ciprofloxacin* or *Gentamicin* did not affect ZOI of *E. coli* when compared to antibiotics alone (*p* > 0.05). However, the combination of 10 µL of LE solution with *Imipenem* or *Ceftazidime* (10 µL solution) resulted in negligible ZOI when compared to a 10 µL solution of antibiotics only (*p* < 0.001); the combination was, therefore, less effective at inhibiting *E. coli* growth compared to using a 10 µL solution of the antibiotics alone (Figure 3).

As for PE, a 10 µL solution added to discs with any of the four antibiotics antagonised their activity, particularly for *Ciprofloxacin* and *Gentamicin*, where ZOI were marginal to none (*p* < 0.001) (Figure 3).

## 3. Discussion

This study aimed to assess the antimicrobial effects of lemon and pomegranate extracts against a range of Gram-positive and Gram-negative bacteria, along with two fungal species. It sought to evaluate the potential of these extracts alone or mixed with antibiotics (*Ciprofloxacin*, *Gentamicin*, *Ceftazidime*, and *Imipenem*) in addressing the challenge of antibiotic resistance. PE exhibited prominent antimicrobial effects, with LE demonstrating antimicrobial properties to a reduced degree. PE showed greater or similar antibacterial potential to *Ciprofloxacin*, *Gentamicin*, and *Ceftazidime* against *S. aureus*. Combining antibiotics with either PE or LE (at concentrations of 0.1 g/mL) showed neutral or antagonistic effects, resulting in no impact or a reduction in the activity of some antibiotics.

Our results add to the body of literature reporting anti-bacterial and antifungal effects of PE in vitro [17,20,21,22,23,24,25,26]. We additionally demonstrated that the mode of inhibition of PE against most bacteria was predominantly bactericidal, while LE exhibited bacteriostatic or no mode of inhibition. While it has been suggested that, clinically, the distinction between bacteriostatic and bactericidal agents may not be significant, with both demonstrating efficacy in antibacterial treatments [27,28,29], advantages relating to bactericidal effects have been documented. The bactericidal inhibition could reduce the incidence of resistance to antimicrobials, due to killing pathogens. Conversely, the bacteriostatic inhibition could be a function of the total load of actual antimicrobial compounds present in samples [28]. The acquired findings provide a strong rationale to carry out in vivo research, with the objective of validating and comprehensively understanding the observed antimicrobial effects. Such research would benefit from purifying active antimicrobials from the extracts and testing their values of minimum inhibitory concentration (MIC), which is one of the ultimate criteria for establishing the efficiency of antimicrobials. The growth of *K. pneumoniae* was not yet inhibited by PE, as previously reported in the study of Dey et al. [30]. Given the limited studies looking at the antimicrobial effect of PE on this microorganism, further research is needed before drawing conclusions. 

Our results show that LE (40 µL of 0.1 g/mL stock solution) was not effective against *S. aureus*. This observation contrasts with the effects of LE reported in the literature concerning both Gram-positive and Gram-negative bacteria [31,32]. As the concentrations of lemon extract were not explicitly mentioned in these studies, making direct comparisons may not be conclusive. 

*E. coli* and *S. aureus* are common pathogens that are the leading cause of healthcare-associated infections [33]. *S. aureus* is highly sensitive to *Gentamicin* and *Ciprofloxacin* [34], and our study revealed pronounced antimicrobial activity that is potentially comparable/higher than that in both antibiotics. This outcome is in line with previous results showing greater antibacterial activity for PE than for *Ciprofloxacin* (20 µL of 2 mg/mL stock solution) [35] and similar zones of inhibition to *Gentamicin* [22]. There is, therefore, a need to establish whether PE possesses similar or greater anti-microbial activity than antibiotics in vivo. Given that the extract contained mainly punicalagins (69%), we add to previous evidence supporting the anti-microbial role of punicalagin [35,36], while further clarification of the mechanisms of action is needed.

PE and LE (20 µL of stock solutions at 0.1 g/mL) did not significantly inhibit the growth of *E. coli*, as evidenced by a negligible ZOI. The effect of PE on Gram-negative bacteria was less pronounced than on Gram-positive bacteria, and this could be due to structural differences in the cell wall [36]. Nevertheless, in the first part of our experiment, we reported antimicrobial effects of PE (40 µL of stock solution 0.1 g/mL) on *E. coli*. Therefore, it is possible that greater concentrations of PE might allow detectable effects on Gram-negative bacteria.

Some antagonistic effects were yet observed. In the case of *S. aureus*, a combination of *Imipenem* with either PE or LE reduced the activity of the antibiotic, shown by a statistically lower ZOI compared to *Imipenem* alone. The involvement of both *Imipenem* and phenolic acids in disrupting the bacterial cell wall leading to cell death [37] suggests a potential competition in binding to bacterial cell membrane proteins. Specifically, PE has been documented to disrupt the bacterial cell wall within 2 h of incubation [38], indicating a potentially faster activity compared to *Imipenem*. In the case of E. coli, antagonistic activity of PE (on all antibiotics) and LE (on *Imipenem* and *Ceftazidime*) was noted. Albeit not in line with previous findings reporting synergistic effects of PE and antibiotics on *E. coli* [22] and LE [39], antagonistic effects of polyphenols have been previously reported [21]. A plausible explanation of the controversial effect lies in phenolic compounds acting like a “double-edge sword”. A study involving casticin (a type of flavonol) demonstrated that it was antagonistic to antibiotics at a concentration ratio of 1:3 (antibiotic to casticin, *v*/*v*), while it was synergistic at lower concentrations of this flavonol. The mechanisms of actions and agents involved in this phenomenon remain elusive, primarily attributed to the antioxidant activity of polyphenols at low concentrations and their pro-oxidant effects at higher concentrations [40]. This outcome may have significant implications in food–drug interactions. While the doses of pomegranate utilised in this study may not correspond to the typical intake levels from pomegranate juice or fruit consumed in humans [41], an analysis of lemon juice revealed that the concentration of eriocitrin is 6%, a noteworthy finding given the widespread use of lemon juice as a home remedy during flu infections [42]. If the antagonistic effects of PE and LE are confirmed at high concentrations, a caution may be warranted against the consumption of elevated doses of pomegranate and lemon during acute bacterial infections that require taking antibiotics. These extracts may still yet be recommended overall due to their acknowledged antibacterial properties. In vivo studies using different concentrations of extracts, antibiotics, and combined extracts/antibiotics will establish the concentrations at which synergistic or antagonistic effects are obvious, while ideally clarifying the mechanisms of action involved. For instance, *Ciprofloxacin* resistance is primarily attributed to mutations in type II topoisomerases [43]; punicalagin, has been shown to target topoisomerase II in vitro [44], which could constitute an important mechanism to be examined in animal studies and translate to human research.

Comparable zones of inhibition were shown when PE was added to *Ciprofloxacin* and *Ceftazidime* against *S. aureus* and when LE was added to *Ciprofloxacin* and *Gentamicin* against *E. coli*. Despite not exerting synergistic effects, such a combination of antibiotics may hold significance in the context of antibiotic resistance. Antioxidants have been shown to reduce antibiotic resistance by mitigating the increased dissemination of resistance plasmids. This process involves the generation of reactive oxygen species (ROS) [45], which can be effectively countered by antioxidants. 

The strengths of this study involve the use of standardised doses of both PE and LE which will facilitate comparison between studies. Additionally, we added a component in testing modes of inhibition for both extracts. With the extracts solely containing polyphenols, we added to the body of literature suggesting their role as anti-microbial agents. A limitation of this study is the use of limited variations in concentrations of extracts/antibiotics, which did not allow the determination of optimal doses that exert beneficial effects in antibiotic activity. Future studies should explore a wider range of concentrations to identify the most effective doses and to evaluate their impact on antibiotic efficacy more comprehensively.

## 4. Materials and Methods

### 4.1. Preparation of Extracts

Dry powders of PE (POMANOX^®^ P30) and LE (WELLEMON^®^) extracted from whole edible fruits were provided by Euromed S.A (Barcelona, Spain). PE contains 76.3% of ellagitannins (75% punicalagins and 1.3% ellagic), while LE contains 12% of eriocitrin. Analysis was conducted using the HPLC method. 

Powders of both extracts were dissolved in distilled water to make up concentrations of 0.1 g/mL. The solutions were then filtered with disposable microbiological filters (with pore size 0.45 µm) to remove any debris and reduce the bulk of microbes. The extract solutions were stored in the refrigerator at 4 °C if not used immediately. 

It is known that pH can contribute to antimicrobial activity. Our unpublished lab data indicated that the antimicrobial effects of various drinks, including grape juice, pomegranate juice, and cranberry drink (with pH values ranging from 2.5 to 3.55), did not correlate with their pH levels. Additionally, control solutions with equivalent pH values, prepared using acetic acid in water did not exhibit significant antimicrobial differences.

### 4.2. Testing Antibacterial and Antifungal Activities of PE and LE

#### 4.2.1. Microbial Strains

Freshly grown bacterial cultures of *B. cereus* (MCIMB 9373), *E. coli* (NCTC 9001), *K. oxytoca* (ATCC 15764), *K. pneumoniae* (40602), *P. aeruginosa* (NCTC 6749), MRSA (NCTC 13552), *S. epidermidis* (NCTC 11047), and fungal cultures of *C. albicans* and *C. glabrata* were used. Bacteria were cultivated on nutrient agar plates overnight (at 30 °C for *B. cereus* and *S. epidermidis*, and 37 °C for other bacteria). Yeast species were cultured over two days at 37 °C on malt extract agar.

#### 4.2.2. Sensitivity Testing

Sterile paper discs (6 mm in diameter) were loaded with 40 μL stock solution of either PE or LE (at a concentration 0.1 g/mL); 20 μL of each extract (or autoclaved water) was first loaded, then dried for 10 min at 50 °C, with the procedure repeated twice. If not used immediately, the loaded discs were stored in the refrigerator for later usage. Sensitivity tests were performed as previously described [46].

In brief, 1 mL of saline was added in each plastic bijou. One to three colonies of the bacteria were taken with a wooden skewer and released into the saline. Sensitivity testing was achieved by comparison with the 0.5 McFarland standard, followed by diluting 20 μL of the bacteria sample in 1 mL of saline, and swabbing onto Mueller–Hinton agar. For fungi, the preparation procedures were similar (except distilled water rather than saline was used and no further dilution of the initial suspension was required), and the suspensions were swabbed onto malt extract agar for sensitivity testing. 

Discs loaded with PE, LE, and water (control) were placed onto plates (swabbed with microbial strains) and pressed slightly to the agar. As a control, discs with autoclaved water were placed onto each plate. Plates were then incubated overnight at the permissive temperature for each strain. Antibacterial and antifungal activity were assessed as diameters of ZOI, and pictures of the plates were taken against a dark background next to a ruler. 

#### 4.2.3. Modes of Inhibition

For testing mode of inhibition, the previously described simple technique [24] was used. In brief, each ZOI was touched 3 times with sterile forceps or wooden skewers (close to the disc edges) and streaked onto Mueller–Hinton agar (for bacteria) or malt extract agar (for fungi). The plates with *E. coli*, *K. oxytoca*, *K. pneumoniae*, *P. aeruginosa*, MRSA, *C. albicans*, and *C. glabrata* were then incubated at 37 °C overnight, and plates with *B. cereus* and *S. epidermidis* were incubated at 30 °C overnight. No re-growth observed was interpreted as bactericidal inhibition, whereas bacteriostatic effects were concluded based on the restoration of microbial growth. Fungistatic and fungicidal effects were also assessed in a similar manner.

### 4.3. Testing Antibacterial Activity of Antibiotics and/or Extracts

For this experiment, *E. coli* (NCTC 9001) and *Staphylococcus aureus* (NCTR 6571) were used. The bacteria were cultivated on Mueller–Hinton agar plates overnight (at 37 °C). 

#### Preparation of Antibiotic Solutions

Commercial discs with common antibiotics were first immersed in sterile distilled water (10 discs per 1 mL of water), left for 1 h at 25 °C for complete diffusion to make up the following stock solutions of antibiotics: *Ciprofloxacin* 50 ng/µL, *Imipenem* 100 ng/µL, *Gentamicin* 100 ng/µL, and *Ceftazidime* 300 ng/µL. Discs were dried at room temperature for at least 20 min. The antibacterial activity of PE and LE was then evaluated alongside 2 different concentrations of antibiotics by loading volumes of 10 µL and 20 µL of antibiotic solutions onto sterile blank discs. Two different concentrations of PE and LE extracts were used by loading a volume of 10 µL and 20 µL extract solutions (0.1 g/mL) onto discs. Different combinations of antibiotics and/or extracts were used, as shown in Table 1. 

Cells of *S. aureus* and *E. coli* bacteria were prepared and used for sensitivity tests, as described in Section 2.2.2. Plates with swabbed bacteria were then loaded with discs. Each plate contained 4 discs (4 antibiotics) or 2 discs in the case of control (PE and LE with no antibiotics). All experiments were performed in triplicate. Petri dishes were then incubated overnight at 37 °C. Pictures of the dishes were taken and diameters of ZOI were then measured, as previously described (Section 4.2.2).

### 4.4. Statistical Analyses

Analysis was carried out using SPSS (v.29, Chicago, IL, USA). Values were expressed as mean (SD) unless otherwise stated. When possible, one-way ANOVA was used to investigate the effects of various samples on zones of inhibitions. Post hoc comparisons were conducted using the Tukey test. Statistical significance was determined at a significance level of *p* < 0.05.

## 5. Conclusions

In conclusion, our study provides additional insights into the antibacterial and antifungal attributes of PE and LE, highlighting predominantly bactericidal effects of PE and bacteriostatic effects of LE. PE manifested more pronounced effects than certain antibiotics, whereas some antagonistic effects to the activity of antibiotics were reported when combined with the extracts. Therefore, prioritising in vivo investigations, commencing with animal studies, and progressing to human trials, becomes imperative to establish the efficacy of these extracts to combat antibiotic resistance.

## Figures and Tables

**Table 1:** Concentrations of antibiotics in stock solutions and their volumes loaded onto blank discs.

Assays	Loading Volumes and Concentrations per Disc
A1	20 µL of antibiotic solution: • *Ciprofloxacin =* 50 ng/µL • *Gentamicin* = 100 ng/µL • *Imipenem* = 100 ng/µL • *Ceftazidime* = 300 ng/µL
A2	10 µL of antibiotic solution (*Ciprofloxacin* = 50 ng/µL; *Gentamicin* = 100 ng/µL; *Imipenem* = 100 ng/µL; *Ceftazidime* = 300 ng/µL) + 10 µL of water
A3	10 µL of antibiotic (*Ciprofloxacin* = 50 ng/µL; *Gentamicin* = 100 ng/µL; *Imipenem* = 100 ng/µL; *Ceftazidime* = 300 ng/µL) + 10 µL of PE (0.1 g/mL)
A4	20 µL of PE (0.1 g/mL)
A5	10 µL of PE (0.1 g/mL) + 10 µL of water
A6	10 µL of antibiotic (*Ciprofloxacin* = 50 ng/µL; *Gentamicin* = 100 ng/µL; *Imipenem* = 100 ng/µL; *Ceftazidime* = 300 ng/µL) + 10 µL of LE (0.1 g/mL)
A7	20 µL of LE (0.1 g/mL)
A8	10 µL of LE (0.1 g/mL) + 10 µL of water

## Data Availability

All data is available in Supplementary Materials S1–S3.

## Supplementary Materials

The following supporting information can be downloaded at: https://www.mdpi.com/article/10.3390/ijms25136943/s1.
