# Supplementary material for: Antimicrobial Potential of Pomegranate and Lemon Extracts Alone or in Combination with Antibiotics against Pathogens
Source: Int J Mol Sci. 2024 Jun 25;25(13):6943. doi: 10.3390/ijms25136943 (PMC11241309; doi:10.3390/ijms25136943)
Supplement: Supplementary file 1 [file ijms-25-06943-s001.zip › Supplementary material 1.pdf]

Supplementary material 1: Antimicrobial effects of lemon extract and pomegranate extracts.

	ZOI (diameter in mm)				
	**Disc 1**	**Disc 2**	**Disc 3**	**Mean (SD)**	**MOI**
Bacteria					
B. cereus					
LE	8	8	8	8 (0)	Bactericidal
PE	18	16	16	16.67 (1.15)	Bactericidal
E. coli					
LE	nil	nil	nil	N/A	N/A
PE	14	14	12	13.33 (1.15)	Bacteriostatic
K. oxytoca					
LE	nil	nil	nil	N/A	N/A
PE	12	12	12	12 (0)	Bactericidal
K. pneumoniae					
LE	nil	nil	nil	N/A	N/A
PE	nil	nil	nil	N/A	N/A
MRSA					
LE	10	8	10	9.33 (1.15)	Bacteriostatic
PE	28	26	26	26.67 (1.15)	Bactericidal
P. aeruginosa					
LE	nil	nil	nil	N/A	N/A
PE	18	18	20	18.67 (1.15)	Bactericidal
S. epidermidis					
LE	nil	nil	nil	N/A	N/A
PE	12	12	12	12 (0)	Bacteriostatic
Fungi:					
C. albicans					
LE	nil	nil	nil	N/A	N/A
PE	12	14	14	13.33 (1.15)	Fungistatic
C. glabrata					
LE	8	8	8	8 (0)	Fungistatic
PE	16	16	16	16 (0)	Fungistatic

ZOI: zones of inhibition; MOI: modes of inhibition

N/A: Not available
