# Supplementary material for: Antimicrobial Potential of Pomegranate and Lemon Extracts Alone or in Combination with Antibiotics against Pathogens
Source: Int J Mol Sci. 2024 Jun 25;25(13):6943. doi: 10.3390/ijms25136943 (PMC11241309; doi:10.3390/ijms25136943)
Supplement: Supplementary file 1 [file ijms-25-06943-s001.zip › Supplementary material 2.pdf]

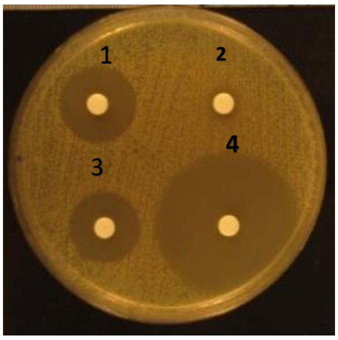

20 µL antibiotic (1)

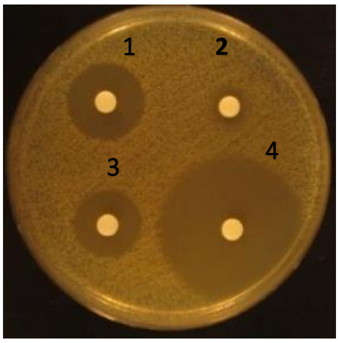

20 µL antibiotic (2)

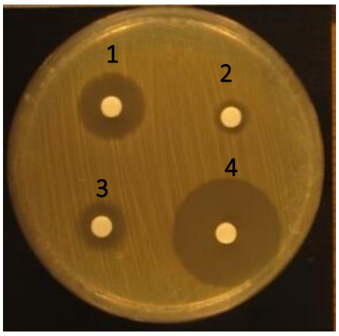

20 µL antibiotic (3)

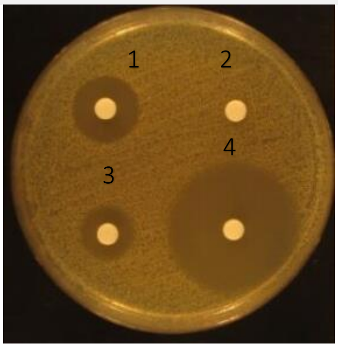

10 µL antibiotic (1)

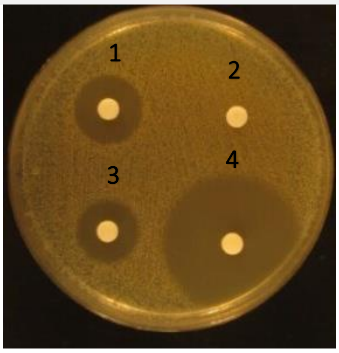

10 µL antibiotic (2)

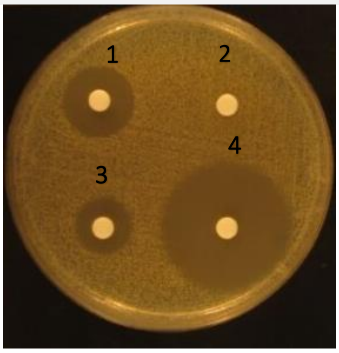

10 µL antibiotic (3)

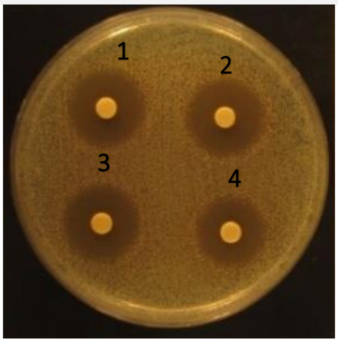

10 µL antibiotic + 10 µL PE (1)

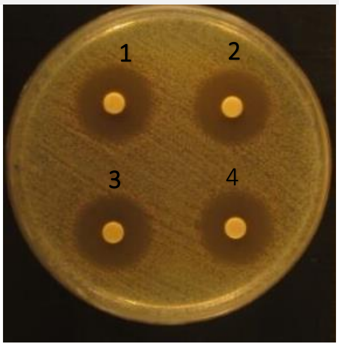

10 µL antibiotic + 10 µL PE (2)

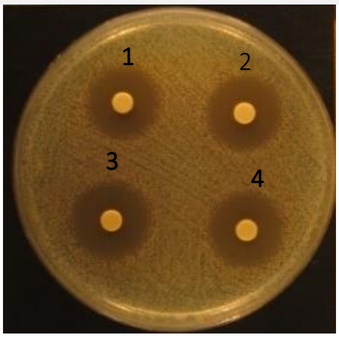

10 µL antibiotic + 10 µL PE (3)

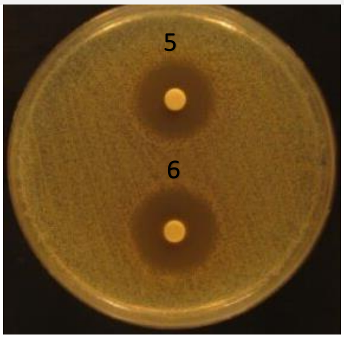

PE (1)

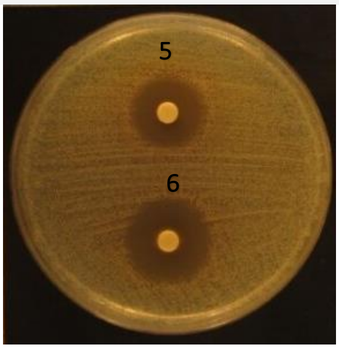

PE (2)

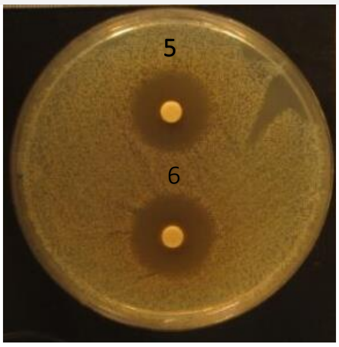

PE (3)

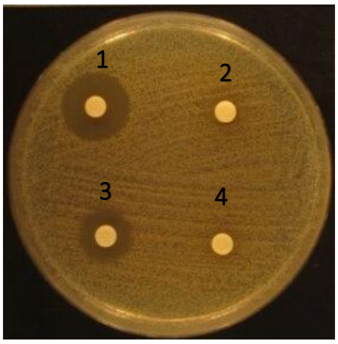

10 µL antibiotic + 10 µL LE (1)

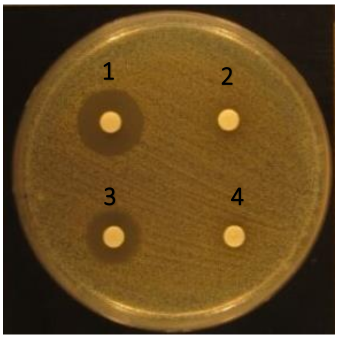

10 µL antibiotic + 10 µL LE (2)

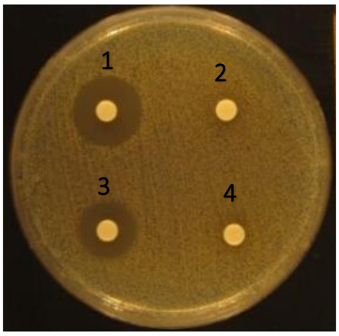

10 µL antibiotic + 10 µL LE (3)

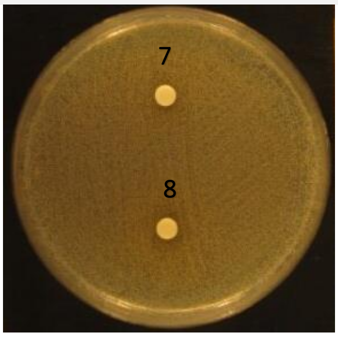

LE (1)

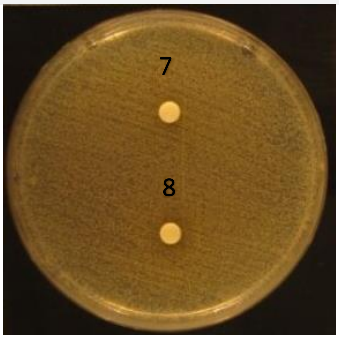

LE (2)

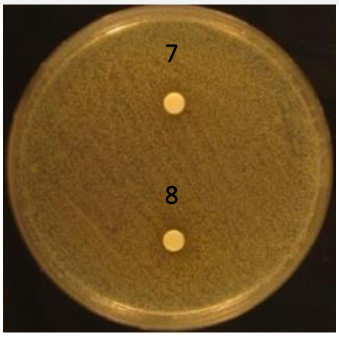

LE (3)

Supplementary material 2: Mueller-Hinton agar plates for the antibacterial activity of PE and LE against S. aureus.

PE: pomegranate extract; LE: lemon extract

Numbers represent 1) Ciprofloxacin (50 ng/µL); 2) Gentamicin (100 ng/µL); 3) Imipenem (100 ng/µL); 4) Ceftazidime (300 ng/µL); 5) 20 µL PE (0.1 g/mL stock solution); 6) 10 µL PE (0.1 g/mL stock solution); 7) 20 µL LE (0.1 g/mL stock solution); 8) 10 µL PE (0.1 g/mL stock solution). All experiments were performed in triplicate.
